# Otolaryngology Surgeon‐Scientists: The Road Less Traveled

**DOI:** 10.1002/oto2.70228

**Published:** 2026-04-20

**Authors:** Tonya Aaron, Janice Huang, Soumil Prasad, Pavan S. Krishnan, Garrett Forman, Lucienna Wolf, Eric Sokhn, Taylor Kring, Bryan Souza, Christine T. Dinh, Elizabeth J. Franzmann, Xue Z. Liu

**Affiliations:** ^1^ Department of Otolaryngology–Head & Neck Surgery University of Miami Miller School of Medicine Miami Florida USA; ^2^ Department of Otolaryngology‐Head & Neck Surgery Albert Einstein College of Medicine Bronx New York USA

**Keywords:** education, residency training, surgeon‐scientist

## Abstract

**Objective:**

Surgeon‐Scientists in Otolaryngology‐Head and Neck Surgery (OHNS) are critical to advancing clinical care through scientific innovation. However, extended training duration and challenges in research funding acquisition continue to hinder career advancement.

**Study Design:**

Cross‐sectional cohort study.

**Setting:**

Analysis of publicly available NIH RePORTER data and national survey of NIH‐funded OHNS faculty across US academic medical institutions. Survey data was collected between October and December 2024.

**Methods:**

We identified NIH‐funded OHNS faculty as of September 2024 using the NIH RePORTER database. We examined time to initial R01‐equivalent funding and grant diversity by degree types (MD, PhD, or dual‐degree). A survey was administered to the OHNS faculty to evaluate their training experiences, research productivity, and funding mechanisms.

**Results:**

PhD faculty comprised the largest proportion of NIH‐funded OHNS investigators (52%) and had greater grant diversity than MD and dual‐degree counterparts (*P* < .0001). Furthermore, PhDs secured R01‐equivalent grants earlier than MDs and dual‐degree holders (7.5 vs 9.5 years posttraining, *P* = .04). However, MD and dual‐degree faculty were more likely to obtain K awards and transition to R01 funding (*P* < .0001). Participation in T32/R25 residency programs was associated with a 2.5‐year reduction in time to R01‐equivalent grant (*P* = .037). Basic science researchers were 4 times more likely to obtain K awards (*P* = .04).

**Conclusion:**

While OHNS PhD faculty attain early and diverse funding, structured training programs and engagement in basic science research significantly enhance MD‐degree faculty funding success. This study highlights institutional and training‐level strategies that reduce the time to research independence and strengthen the OHNS surgeon‐scientist pipeline.

Otolaryngology‐head and neck surgery (OHNS) surgeon‐scientists serve as a bridge between clinical practice and scientific innovation by advancing patient care and improving outcomes through their dual expertise.^1^ Studies suggest that while many trainees engage in research during residency, fewer continue as independently funded investigators.^2^ In fact, OHNS ranked 12th out of 18 medical specialties receiving NIH funding in total inflation‐adjusted funding amount.^3^ Despite these challenges, surgeon‐scientists have made significant contributions to the advancement of head and neck cancer treatment, hearing restoration, and the management of airway diseases. Furthermore, those with dedicated research training are more likely to publish, secure grants, and assume leadership roles.^4^, ^5^, ^6^

Between 2009 and 2019, there was a decline in OHNS NIH‐funded projects, with funding disproportionately concentrated among a small number of institutions and principal investigators.^3^ Thus, several initiatives were developed to support the growth of surgeon scientists. NIDCD‐funded research‐track residencies, MD/PhD programs, and foundation research grants (such as the AAO‐HNSF CORE grants) have been shown to facilitate early career engagement in research.^7^, ^8^ Studies indicate that recipients of these grants are more likely to secure NIH K‐awards and transition into academic careers.^7^ Furthermore, structured mentorship and protected research time during residency have been associated with higher scholarly productivity and long‐term retention in academic medicine.^9^, ^10^

Our research seeks to characterize the current landscape of NIH‐funded academic OHNS faculty, comparing surgeon‐scientists (MD or dual degree) with PhD researchers. We aim to determine whether differences exist between these groups in securing career development awards and subsequent research grants. This analysis will illuminate the unique opportunities and challenges faced by surgeon‐scientists pursuing funded research careers in OHNS. Findings from this study will inform the development of more effective training frameworks and institutional policies to support and retain OHNS surgeon‐scientists. By understanding these critical factors, we can foster an environment that promotes scientific inquiry and innovation, ensuring continued progress and meaningful contributions to the field of OHNS.

## Methods

In this cross‐sectional study, 288 principal investigators with OHNS department appointments and active funding were identified in the NIH Research Portfolio Online Reporting Tools database (NIH RePORTER) in September 2024. Graduate students, postdoctoral fellows, residents, and instructors were excluded from the analysis, and 251 faculty with OHNS appointments were included in this study. Demographic information (sex, degree type, current academic rank, specialty, year of training completion) was extracted from faculty pages on institution websites, LinkedIn, Doximity, and publicly available curriculum vitae. Faculty NIH grant history was extracted from the NIH RePORTER database.

To identify factors supporting otolaryngologists in becoming principal investigators on R01 grants, we developed a 31‐question de‐identified survey using Google Forms assessing demographics and research‐related career trajectories of current OHNS MD, MD/PhD, and MD/MPH faculty. Faculty were identified from the NIH RePORTER database. Of the 110 eligible individuals, 102 were emailed, and 46 responded (45%). The survey was conducted between October and December 2024, with biweekly reminders. This study was deemed IRB exempt.

The survey collected demographic variables, including sex, race, first‐generation status, degree type, academic rank, specialty, and career stage at which participants had children. Research‐related data included: publication count at each career stage, field of research, and sources of non‐NIH funding. None of the survey participants reported 100% protected research time, indicating that they are still clinically active.

### Statistical Analysis

#### NIH RePORTER Database Statistics

Demographic representation and grant diversity were compared between PHD faculty and MD degree faculty using chi‐square analysis. To further assess differences in grant diversity, a post hoc pairwise chi‐square test with Bonferroni‐adjustment was performed.

To evaluate differences in the time interval (years) from training completion to key research milestones, Kruskal‐Wallis tests were conducted. Training completion was defined as the last year of fellowship or residency for MD faculty and postdoctoral fellowship for PhD faculty. The two primary research outcomes assessed were (1) acquisition of a career development award (K08, K23, K24, K99/R00) and (2) receipt of an R01‐equivalent research grant. R01 equivalent grants were defined according to NIH criteria and included DP1, DP2, DP5, R37, R56, RF1, RL1, and U01 grants. In addition, Kruskal‐Wallis tests were used to examine differences in the interval (years) between receiving a K award and transitioning into an R award, and the K‐to‐R transition rate (K‐to‐R transition) as previously defined by Silvestre et al.^11^ The K‐to‐R transition rate was defined as the proportion of individuals within each group who obtained a K award and subsequently secured a R01 grant. If a significant difference among degree types was detected, Dunn's multiple comparison tests were used for post‐hoc analysis, with PhD faculty as the reference group.

Pearson chi‐square tests were used to determine if there was an association between degree type and research outcomes. Furthermore, univariate linear regression was conducted to determine the likelihood of achieving each research milestone based on degree type.

#### Survey Statistics

Stepwise linear regression was performed to identify training factors (publications, participation in research training programs) or demographic variables (degree type, sex, first‐generation status, specialty, and career stage at first childbirth) associated with the time required for surgeon scientists to become principal investigators of R01‐equivalent grants.

To assess factors influencing key research milestones, stepwise logistic regression was used to evaluate the impact of demographic characteristics, training background, research field, or non‐NIH funding sources on the likelihood of: (1) obtaining a K08 or K23 career development award, (2) time to acquiring an R01‐equivalent award, and (3) successfully transitions from a K award to an R award (K‐to‐R transition). All statistical analysis was done using SAS software.

## Results

The NIH RePORTER database (National Institutes of Health Research Portfolio Online Reporting Tool Expenditures and Results) identified 251 faculty with OHNS appointments who have active NIH‐funded projects as of September 2024. Among them, PhD faculty represented a majority (52%, n = 131), while MD (n = 51), MD/PhD (n = 49), and MD/MPH (n = 10) surgeon scientists accounted for 20%, 20%, and 4%, respectively (Table 1). Otology/Neurotology (38), Head and Neck (29), and Rhinology/Skull Base (15) are the top 3 represented specialties. There were no significant differences in sex (*P* = 0.45), academic rank (*P* = .31), or subspecialty (*P* = .24) representation across degree types (Table 1).

**Table 1:** Demographics of OHNS Faculty With Active NIH Funding

	MD n = 51	PhD n = 131	MD/PhD n = 49	MD/MPH n = 10	Other n = 10*	*P* value
Sex (female) n (%)	15 (29)	50 (38)	13 (27)	5 (50)	4 (40)	.45
Academic rank n (%)						.31
Professor	30 (59)	53(40)	27 (55)	4 (40)	2 (20)	
Associate Professor	13 (25)	39(30)	11 (22)	4 (40)	3 (30)	
Assistant Professor	8 (16)	35(27)	10 (20)	2 (20)	4 (40)	
Other		4(3)	1 (2)		1 (10)	
Subspecialty n (%)						.24
General	4 (8)		2 (4)	1 (10)		
FPRS	0 (0)		1 (2)	0 (0)		
Rhinology/skull base	9 (18)		5 (10)	1 (10)		
H&N	13 (25)		14 (29)	2 (20)		
Laryngology	5 (10)		3 (6)	1 (10)		
Otology/Neurotology	13 (25)		23 (47)	2 (20)		
Sleep Medicine	1 (2)		0 (0)	1 (10)		
Pediatrics	6 (12)		1 (2)	2 (20)		
						

Chi‐square analysis identified associations between degree type and sex, academic rank, or subspecialty. Other group (5 AuD/PhDs, MBA/PhD, MPH/PhD, PhD CCC‐SLP, Engineer, MD/DVM).

**Figure 1:**
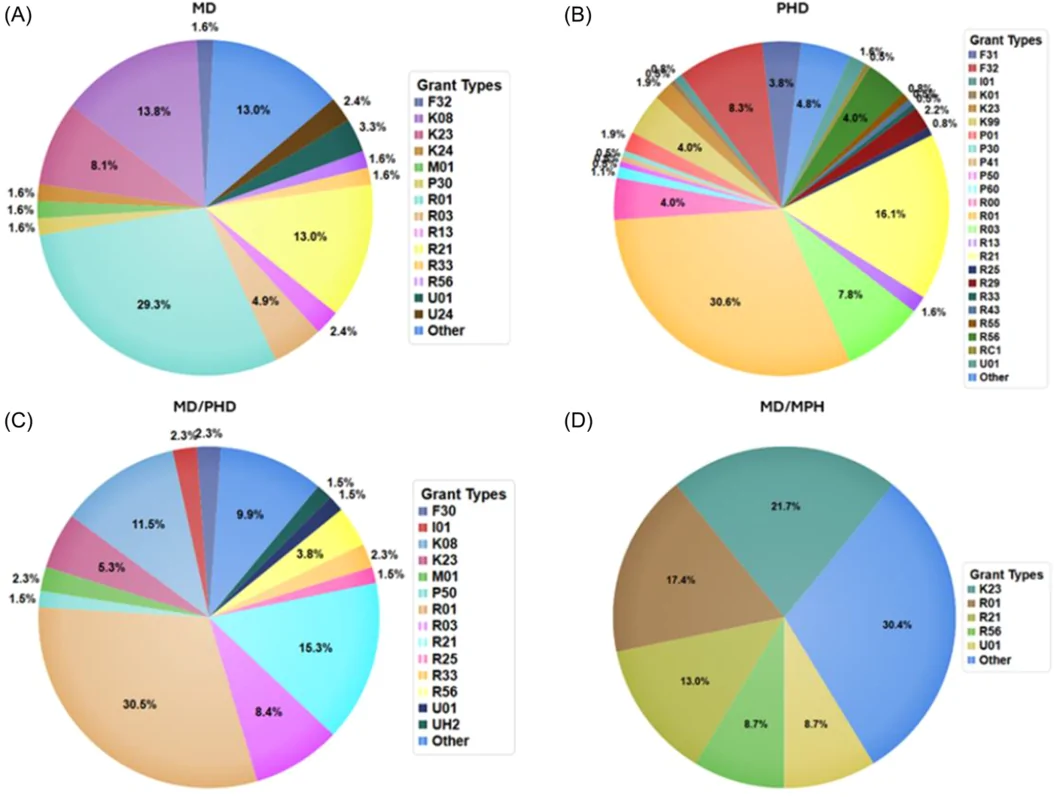
Grant diversity. Chi‐square tests compared grant diversity across degree types. Grant type must be held by more than one person. See Table 1 for n of each group.

Next, we analyzed grant diversity among OHNS faculty in the database to assess potential disparities in funding opportunities. The number of distinct grants varied by degree type: PhD (42), MD (30), MD/PhD (27), MD/MPH (12). Chi‐square analysis showed significant differences in grant diversity (*χ* ^2^(41) = 75.8, *P* < .0001), with PhD faculty securing the most unique grants (Figure 1). Pairwise comparisons confirmed greater grant diversity among PhD faculty compared to all MD‐degree groups (MD vs PhD, *P* < .0001; MD/PhD vs PhD, *P* < .0001; MD/MPH vs PhD, *P* = .0008). In contrast, no significant differences in grant diversity were observed between MD‐degree groups (MD vs MD/PhD, *P* = .62; MD vs MD/MPH, *P* = .33; MD/PHD vs MD/MPH, *P* = .04). A Bonferroni‐adjusted significance threshold of *P* < .0083 was applied. While general otolaryngologists (n = 7) were less represented, they had the highest grant‐to‐investigator ratio (3.14), compared to Otology/Neurotology (2.52), Head and Neck (2.90), and Rhinology/Skull Base (2.63) (data not depicted). For subsequent analysis, MD/PhD and MD/MPH faculty are grouped as dual‐degree faculty.

Although the R01 research project grant is not the sole mechanism for establishing an independent investigator research career in academic medicine, it often provides the funding support to initiate the infrastructure and research team essential for career progression. Using the NIH RePORTER data and publicly available training completion data, we sought to determine if there were differences in the acquisition for career milestone awards (K08, K23, K99/R00) and grants (R01‐equivalent), the time interval between training completion and achievement of the previously mentioned career milestones, and successful transition from K award to R award (K to‐R transition) (Table 2).

**Table 2:** Funding Acquisition and K‐to‐R Transition Rates

	MD n = 51	PhD n = 131	Dual‐Degree n = 59	*P* value
Average years to K award (% with award)	3.7(55%)	4.9(19%)	3.7(51%)	.81
Average years to R01‐equivalent award (% with award)	9.5(71%)	7.4(75%)	9.4(75%)	**.04**
Average time interval for K‐to‐R transition (transition rate)	6.7(37%)	7.1(8.5%)	6.6(31%)	.50

Kruskal‐Wallis tests determined differences in the average time interval (years) between training completion and K or R award acquisition. Two MD/MPHs completed K‐to‐R transition.

There was no significant difference in the time intervals between training completion and K award acquisition (*P* = .81) or K‐to‐R transition (*P* = .50) between degree types. However, PhD faculty obtained R01‐equivalent funding significantly earlier than MD‐degree groups (7.5 vs 9.5 years, *P* = .04), even though there were no differences in frequency of R01 awarded faculty between groups (MD 37/51 (72%), PhD 106/131 (81%), and dual‐degree 43/59 (73%), *P* = .29) (within dual‐degree group MD/PhD 40/49 [82%] and MD MPH 3/10 [30%]).

**Figure 2:**
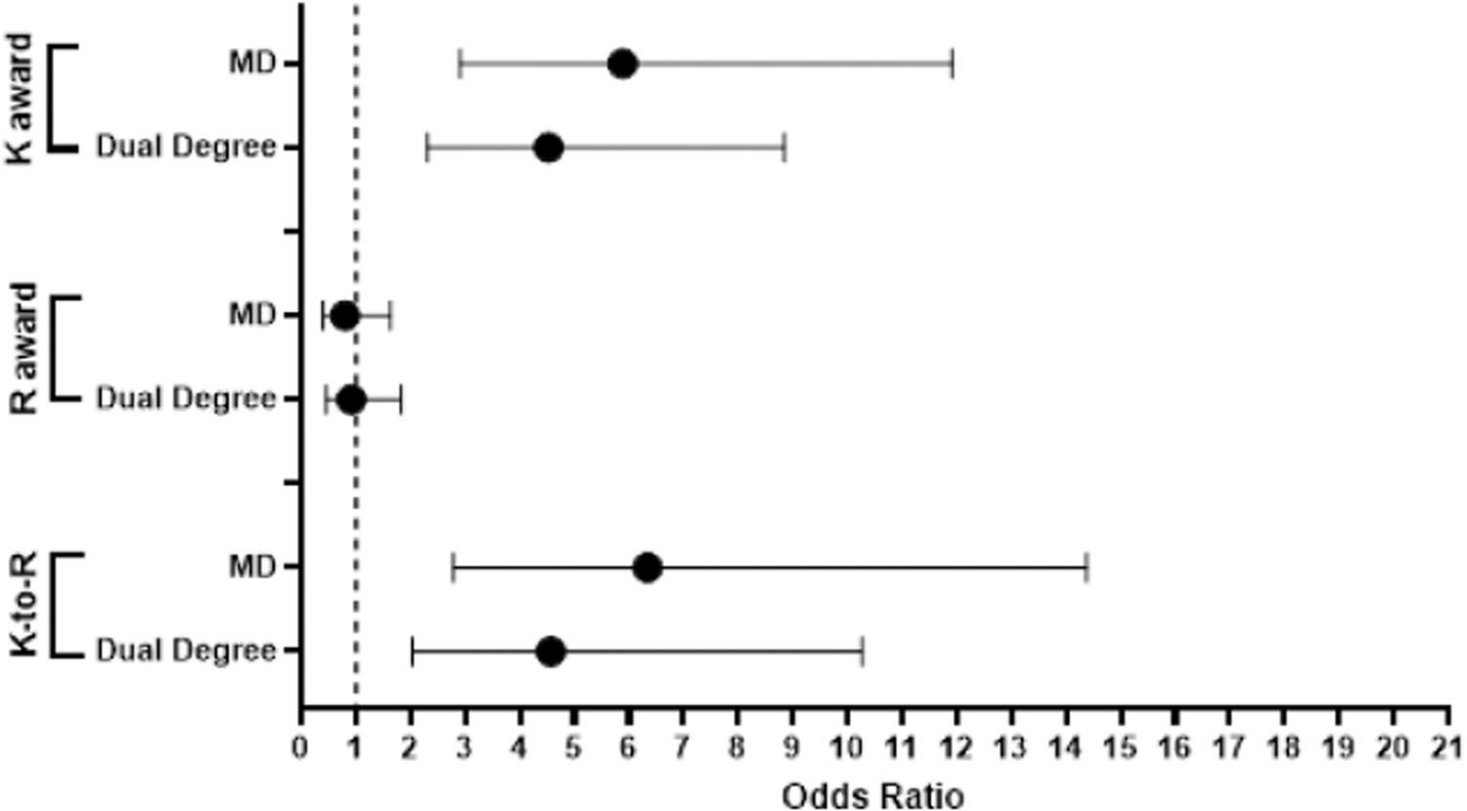
Degree type determines likelihood of grant acquisition. Univariate analysis of award acquisition in surgeon‐scientist subgroups. Data are represented as dots corresponding to odds ratios with 95% CI. CI, confidence interval.

Next, we aimed to identify an association between degree type and the acquisition of K and R awards, and successful K‐to‐R transitions using the data generated from the NIH RePORTER database (Figure 2). Pearson chi‐square tests revealed a significant association between degree type and both K award acquisition (*χ* ^2^(3) = 22.4, *P* < .0001) and K‐to‐R transition rates (*χ* ^2^(3) = 14.7, *P* < .0001). No association was found with R award acquisition (*χ* ^2^(3) = 4.97, *P* = .83).

Univariate analysis showed that compared to PhD faculty, MD (OR: 5.88, 95% CI: 2.91‐11.91, *P* < .0001) and dual‐degree (OR: 4.52, 95% CI: 2.31‐8.84, *P* < .0001) groups were more likely to obtain K awards. Similarly, MD (OR: 6.33, 95% CI 2.79‐14.38, *P* < .0001) and dual‐degree faculty (OR:4.57, 95% 2.035‐10.27, *P* = .0002) were more likely to transition from a K award to an R award compared to PhD faculty. However, there were no differences in the likelihood of acquiring an R award between groups (MD OR: 0.80, 95% CI: 0.39‐1.63, *P* = .54; dual‐degree OR: 0.92, 95% CI: 0.46‐1.82).

Analysis of OHNS faculty revealed that surgeon‐scientists (MD and dual‐degree) were more likely to acquire K awards and successfully transition from a K to an R award than PhD faculty. Therefore, we developed a survey to determine whether participation in research training programs and other research‐related factors (publication productivity, funding type, and field of research) influenced 4 key outcomes: (1) becoming a principal investigator of a career development grant (K08 or K23), (2) acquiring an R01 grant, (3) the time to acquire an R01 equivalent grant after training completion, and (4) successful K‐to‐R transition.

To identify these influencing factors, we developed a comprehensive survey that collected both demographic information (sex, race, first‐generation status, degree type, academic rank, specialty, and career stage at first childbirth) and research‐related data (number of publications at each stage of training, participation in research training programs, field of research, and non‐NIH funding sources) (Table 3).

**Table 3:** Surgeon‐Scientist Demographics and Factors That Influence Career Path

	MD n = 27	MD/PhD n = 14	MD/MPH n = 5	*P* value
Sex (female) n (%)	9 (33)	4 (29)	2 (40)	.87
Race				.43
White	20 (74)	6 (43)	3 (60)	
Black	0 (0)	1 (7)	0 (0)	
Asian	4 (15)	6 (43)	2 (40)	
Multiracial	1 (4)	0 (0)	0 (0)	
Prefer Not To Answer	2 (7)	1 (7)	0 (0)	
First generation n (%)	19 (70)	10 (71)	2 (40)	.38
Children (career stage) n (%)				**.005**
Faculty	6 (22)	1 (7)	4 (80)	
Fellowship	4 (15)	1 (7)	0 (0)	
Residency	15 (56)	6 (43)	0 (0)	
Medical School	2 (7)	1 (7)	0 (0)	
No Children	0 (0)	5 (36)	1 (20)	
Academic rank n (%)				.47
Professor	19 (70)	7 (50)	4 (80)	
Associate Professor	5 (19)	5 (36)	1 (20)	
Assistant Professor	3 (11)	2 (14)	0 (0)	
Subspecialty n (%)				.24
General	2 (7)	1 (7)	0 (0)	
FPRS	1 (4)	0 (0)	0 (0)	
Rhinology/Skull Base	4 (15)	0 (0)	0 (0)	
Head and Neck	4 (15)	2 (14)	1 (20)	
Laryngology	5 (18)	1 (7)	0 (10)	
Otology/Neurotology	10 (37)	9 (64)	3 (60)	
Sleep Medicine	1 (3)	0 (0)	1 (20)	
Multiple Subspecialties	0 (0)	1 (7)	0 (0)	
Publications n (%)				
Undergraduate				.25
0	20 (74)	8 (57)	3 (60)	
1‐5	7 (26)	4 (29)	2 (2)	
5‐10	0 (0)	0 (0)	0 (0)	
10‐15	0 (0)	0 (0)	0 (0)	
>15	0 (0)	2 (14)	0 (0)	
Medical School				**.02**
0	5 (18)	2 (14)	0 (0)	
1‐5	21 (78)	5 (36)	4 (80)	
5‐10	1 (4)	5 (36)	0 (0)	
10‐15	0 (0)	1 (7)	1 (20)	
>15	0 (0)	1 (7)	0 (0)	
Residency publications				.75
0	1 (4)	0 (0)	0 (0)	
1‐5	6 (22)	6 (43)	4 (40)	
5‐10	7 (26)	2 (14)	0 (0)	
10‐15	6 (22)	6 (28)	2 (40)	
>15	7 (26)	2 (14)	1 (20)	
Fellowship publications				.96
0	3 (11)	2 (14)	1 (20)	
1‐5	15 (56)	7 (50)	3 (60)	
5‐10	5 (18)	3 (21)	0 (0)	
10‐15	3 (11)	1 (7)	1 (20)	
>15	1 (4)	1 (7)	0 (0)	
Research Training Programs n (%)				
T35	1 (4)	1 (7)	0 (0)	.77
MSTP	1 (4)	6 (46)	0 (0)	**.001**
T32	7 (26)	5 (36)	3 (60)	.38
Research n (%)				
Basic Science	11 (41)	9 (64)	0 (0)	**.04**
Translational Science	21 (78)	11 (79)	2 (40)	.19
Clinical Epidemiology	1 (3.7)	4 (29)	2 (40)	**.03**
Clinical Trials	8 (30)	2 (14)	3 (60)	.14
Non‐NIH Funding Sources n (%)				
Veteran Affairs Funding	2 (7.4)	2 (14)	0 (0)	.58
Industry Funding	8 (30)	2 (14)	1 (20)	.53
Institutional Funding	11 (41)	6 (43)	3 (60)	.72
Foundation Funding	9 (33)	4 (29)	1 (20)	.82
Research Outcomes n (%)				
R01 n (%)	19 (70)	10 (71)	2 (40)	**.037**
K08 or K23 n (%)	14 (52)	8 (57)	2 (40)	.80
K‐to‐R transition n (%)*	13 (48)	8 (57)	2 (40)	.77
Time to R01‐equivalent (SD)**	2.98 (2.9)	2.4 (3.14)	1.9 (2.07)	.48

Chi‐square analyses determined if there were differences in demographic or research‐related factors between degree types. Factors found to have significant differences between degree types are in bold.

Chi‐square analysis revealed several significant associations between degree type and faculty characteristics, training experiences, and research outcomes. The timing of parenthood differed significantly by degree type, with MD/MPH faculty more frequently having children during faculty stage (*P* = .005), whereas the majority of MD and MD/PhD faculty had children during residency. Publication output during medical school also differed by degree type (*P* = .02). As expected, MD/PhD faculty were more likely to participate in medical scientist training programs (MSTP) (*P* = .001), and basic science (*P* = .04). Additionally, MD and MD/PhD faculty were more likely to have obtained R01 grants compared to their MD/MPH counterparts (*P* = .0037). No significant differences were observed in sex, race, academic rank, K award acquisition, time to R01‐equivalent, or K‐to‐R transition success rates.

Since degree type did not influence the amount of time it took to acquire an R01‐equivalent grant, stepwise linear regression analysis was performed to identify factors during training (such as participation in research training programs and publication productivity) that could influence the time it takes for surgeon‐scientists to an acquire R01‐equivalent grant after training completion. Results showed that participation in a T32/R25 residency track shortened the interval between training completion and obtaining an R01‐equivalent grant by 2.5 years (*β* = 2.51, *t* = −2.18, *P* = .037).

Stepwise logistic regression analysis showed that the number of medical school publications negatively correlated with R01 grant acquisition (OR:0.37, CI: 0.143‐0.968, *P* = .043). Interestingly, no demographic or training factors were found to predict the acquisition of a K08 or K23 award (*P* = .66) or K‐to‐R transition (*P* = .88).

Since training factors did not predict career development award acquisition, we aimed to determine whether participation in a specific research field or having a non‐NIH funding source could predict research career outcomes. Stepwise logistic regression analysis revealed that basic science surgeon‐scientist researchers are approximately four times more likely to acquire a career development award (K08 or K23) compared to non‐basic science researchers (OR: 3.73, CI: 1.08‐12.9, *P* = .04). In our survey cohort, the majority (64%) of the MD/PhD surgeon‐scientists are engaged in basic science research, whereas 41% of MD and 0% of MD/MPH surgeon‐scientists participate in basic science research.

## Discussion

Historically, residency research tracks vary in their ability to influence the career trajectories of OHNS surgeon‐scientists. Since 1974, T32‐funded programs provided 1 to 2‐year structured research training and financial support.^12^ In 2020, the National Institute of Deafness and Other Communication Disorders (NIDCD) transitioned to the more flexible R25 mechanism so institutions could tailor their research experience for trainees to reflect the duality of the surgeon scientist career with research and clinical training with an 80/20 time split.^13^ Our study shows that participation in T32/R25 residency tracks shortens time to R01‐equivalent funding by 2.5 years, reinforcing the importance of structured research exposure during training.

Consistent with the Kosajaru et al,^13^ we found that surgeon‐scientists are more likely than PhDs to receive career development (K) awards and transition to R funding. However, we did not observe a difference in R01‐equivalent acquisition between degree types.^13^ In this study, PhDs obtained R01‐equivalent grants two years earlier and have greater grant diversity, likely due to having more research training and no clinical obligations. Differences in R01‐equivalent acquisition outcomes were not due to MDs not meeting grant eligibility criteria. Differences between studies may be due to differing inclusion criteria, where Kosaraju et al,^13^ limited their population to OHNS PIs between 2015 and 2021, whereas our population included PIs with active NIH funding as of September 2024.

The prolonged training timeline for surgeon‐scientists remains a barrier for trainees, with the average age of first independent research appointment occurring between the late 30s and early 40s.^8^, ^13^, ^14^ With the average pre‐residency training spanning 10 to 13 years, factors such as gap years before medical school,^8^ prolonged MD‐PhD completion (8.25‐year average),^15^ and research years to improve residency competitiveness^16^ contribute to delays. Notably, our study has found that the number of medical school publications is negatively correlated to R01 grant acquisition, whereas the number of publications during residency has no association with R01 grant acquisition. Similarly, Warren et al^17^ reported that the number of publications during medical school could not predict those that would end up pursuing an academic career in urology. However, residency applicants who matched into urology programs have more publications than unmatched applicants before the implementation of Step 1 pass/fail guidelines. This highlights how medical student research publication is used as a proxy for competitiveness for residency and cannot predict those that will still stay in academic medicine or pursue private practice. Orthopedic residents with higher H‐indexes are more likely to pursue academic careers.^18^ To assess the trajectory of aspiring otolaryngology surgeon‐scientists, retrospective and prospective studies that include metrics like the H‐index of trainees are required to determine predictors of those who are likely to pursue clinician‐scientist careers. Despite these challenges, early structured exposure—such as summer research in college^8^ or medical school accelerated directed pathways^19^—may reduce time in pre‐residency training and encourage trainees to consider a surgeon‐scientist career. Further analysis is required to identify OHNS surgeon‐scientists who participated in accelerated programs and determine if they were supported to pursue a career as surgeon‐scientists.

Although T32/R25 programs offer clear benefits for aspiring OHNS surgeon‐scientists, as of 2024, 11 of 126 OHNS residency programs have an R25 funding mechanism (NIH RePORTER and AAMC). Expanding these opportunities and increasing NIDCD support may bolster early career success.^13^, ^20^ However, the R25 program isn't the only mechanism by which residents can have training in grant writing and research support. In 1985, the Centralized Otolaryngology Research Efforts (CORE) grant was established by the American Academy of Otolaryngology Head and Neck Surgery (AAO‐HNS).^21^ In an assessment of career outcomes of CORE grant recipients, Roy et al. found that 65% of 2010 CORE grant recipients were working in academia as of 2021.^21^ Although all residents are eligible to apply for the CORE grant, most awardees were found to be from top 10 residency programs as recorded by Doximity,^21^ indicating potential disparities in institutional resources and mentor support amongst OHNS residency programs. In addition to participation in a T32/R25 residency research track, experience in basic science research emerged as a strong predictor of career development award acquisition, consistent with previous studies indicating that NIH funding favors mechanistic and translational research over purely clinical or epidemiological studies.^3^, ^11^, ^22^ Our survey results showed that MD/PhD faculty are more likely to conduct basic science research, while MD faculty are more likely to engage in clinical trials. A survey of surgeon‐scientists who conduct basic science research found that those who perceived their department as prioritizing basic science were funded more than those who felt their departments did not prioritize basic science.^22^ Furthermore, basic scientists submitted federal grants more frequently than clinical researchers; however, those who submitted greater than 5 grants were likely to be funded regardless of the research focus.^22^ These findings indicate that grant writing support and persistent grant application may reduce the federal funding disparity seen between basic and clinical researchers.

The challenges faced by OHNS surgeon‐scientists reflect broader concerns about the support systems and sustainability of the physician‐scientist workforce. Our findings reinforce the concept that championing early and structured research opportunities for trainees, comprehensive funding mechanisms, and institutional support is crucial for shaping successful research careers and decreasing the amount of time it takes to become an independent researcher.

This study has several limitations. First, only projects specifically listed under an otolaryngology department were extracted from the NIH RePORTER database, meaning that collaborative projects affiliated with other departments or those without a designated department classification were not captured. As a result, the findings may not fully represent the breadth of research conducted by OHNS faculty. Second, NIH RePORTER does not provide data on unsuccessful applications, limiting our ability to analyze success rates in obtaining NIH funding. This omission prevents an assessment of funding competitiveness between degree types. Lastly, this study did not account for trends over time, despite changes in research training opportunities and structured pathways for OHNS trainees. As institutions continue to refine their research track offerings and medical schools shorten their curriculum, future studies should evaluate how these changes impact long‐term research engagement and funding outcomes.

## Author Contributions

**Tonya Aaron**, MD, PhD, project design, literature review, data and statistical analysis, manuscript writing and editing; **Janice Huang**, project design, literature review, data analysis, manuscript writing, and editing; **Soumil Prasad**, project design, manuscript writing, and editing; **Pavan S. Krishnan, MD**, project design, manuscript writing and editing; **Garrett Forman**, project design, literature review, manuscript writing, and editing; **Lucienna Wolf**, project design, literature review, and data analysis; **Eric Sokhn**, project design, literature review, and data analysis; **Taylor Kring**, project design, literature review, and data analysis; **Bryan Souza**, project design and data analysis; **Christine T. Dinh**, MD, project design, manuscript writing, and editing; **Elizabeth J. Franzmann**, MD, project design, manuscript writing, and editing; **Xue Z. Liu**, MD, PhD FACS, project design, manuscript writing, and editing.

## Disclosures

### Competing interests

None.

### Funding source

NIDCD R25 DC020726 to XL. TA, LW ‐R25 medical students; PSK – R25 resident.
